# (C)overt attention and visual speller design in an ERP-based brain-computer interface

**DOI:** 10.1186/1744-9081-6-28

**Published:** 2010-05-28

**Authors:** Matthias S Treder, Benjamin Blankertz

**Affiliations:** 1 Berlin Institute of Technology, Machine Learning Laboratory, Berlin, Germany; 2 Donders Institute for Brain, Cognition and Behaviour, Centre for Cognition, Nijmegen, The Netherlands; 3 Fraunhofer FIRST, Berlin, Germany

## Abstract

**Background:**

In a visual oddball paradigm, attention to an event usually modulates the event-related potential (ERP). An ERP-based brain-computer interface (BCI) exploits this neural mechanism for communication. Hitherto, it was unclear to what extent the accuracy of such a BCI requires eye movements (overt attention) or whether it is also feasible for targets in the visual periphery (covert attention). Also unclear was how the visual design of the BCI can be improved to meet peculiarities of peripheral vision such as low spatial acuity and crowding.

**Method:**

Healthy participants (N = 13) performed a copy-spelling task wherein they had to count target intensifications. EEG and eye movements were recorded concurrently. First, (c)overt attention was investigated by way of a target fixation condition and a central fixation condition. In the latter, participants had to fixate a dot in the center of the screen and allocate their attention to a target in the visual periphery. Second, the effect of visual speller layout was investigated by comparing the symbol Matrix to an ERP-based Hex-o-Spell, a two-levels speller consisting of six discs arranged on an invisible hexagon.

**Results:**

We assessed counting errors, ERP amplitudes, and offline classification performance. There is an advantage (i.e., less errors, larger ERP amplitude modulation, better classification) of overt attention over covert attention, and there is also an advantage of the Hex-o-Spell over the Matrix. Using overt attention, P1, N1, P2, N2, and P3 components are enhanced by attention. Using covert attention, only N2 and P3 are enhanced for both spellers, and N1 and P2 are modulated when using the Hex-o-Spell but not when using the Matrix. Consequently, classifiers rely mainly on early evoked potentials in overt attention and on later cognitive components in covert attention.

**Conclusions:**

Both overt and covert attention can be used to drive an ERP-based BCI, but performance is markedly lower for covert attention. The Hex-o-Spell outperforms the Matrix, especially when eye movements are not permitted, illustrating that performance can be increased if one accounts for peculiarities of peripheral vision.

## Background

A brain-computer interface (BCI) based on event-related potentials (ERPs) exploits the fact that the neural processing of a stimulus can be modulated by attention. In particular, attention to an event can enhance the positive and negative peaks of the ERP time-locked to this event. ERP-based BCIs attempt to detect these modulations to infer the stimulus that the user intended to choose. Often, the BCI is implemented in an oddball paradigm, wherein rare target events are interspersed with frequent nontarget events. The first such device was introduced by Farwell and Donchin [1]. The authors coined the name P300-speller to refer to the fact that classification was mainly based on the P300 component, a large positivity occurring at 300-500 ms post-stimulus upon rare events. A number of variations of the original speller have been developed, and it has also been adapted to non-visual modalities by using auditory [2-6] and tactile [7] stimulation.

The classical Farwell and Donchin speller consists of a 6 × 6 symbol matrix wherein symbols are arranged within rows and columns. We will refer to this kind of speller as the Matrix. Throughout the course of a trial, the rows and columns are intensified (flashed) one after the other in a random order. Since a given target symbol has a chance of 1/6 of being intensified, it constitutes a rare event or oddball. In Farwell and Donchin's study, healthy participants were able to communicate about 12 bits or an equivalent of 2.3 symbols/min. In the past decades, classification techniques improved [8-11] and practical communication rates including feedback of 5.82 symbols/min have been reported [12], but this information throughput is still not competitive when compared to conventional communication means such as speech, typing, or handwriting. In other words, current ERP-based BCIs do not seem to be viable tools for healthy users. Therefore, most BCIs are tailored for use by patients deprived of other means of communication, such as amyotrophic lateral sclerosis (ALS) patients, who suffer from a neurodegenerative disease characterized by a progressive loss of motor function [13-15]. However, most successful implementations, such as the Matrix speller, use a spatial layout wherein the to-be-chosen symbols are placed at different spatial locations. Hitherto, it was unclear whether or not these spellers rely on eye movements. If they do then devices that measure eye movements directly (such as eyetrackers) might outperform visual BCIs. In fact, there is a body of evidence corroborating the efficacy of dwell-time based gaze interaction in a clinical context [16-19]. For healthy users, information throughput of about 10 words per minute has been reported [20].

The present study addresses the question whether an ERP-based BCI is ultimately dependent on eye movements (i.e., overt attention), or whether it can also detect attention deployed in the visual periphery (i.e., covert attention). This is a key issue because, first, the focus of covert attention cannot be inferred from eye movement data. Second, successful communication using ERP-based visual spellers has been demonstrated in ALS patients [21,22], but in progressed stages of the disease, oculomotor control can deteriorate. Dysfunctions in smooth pursuit, slowing of fixations, nystagmus, and abnormalities in Bell's phenomenon have been observed [23-25], as well as corresponding neurophysiological damage to oculomotor nuclei [26]. For patients suffering from these symptoms, communication using eyetrackers might collapse. Using covert attention in the visual periphery, however, is complicated by the fact that peripheral vision is subject to some peculiarities that should be taken into account in the visual design of the BCI. One of these peculiarities is the decline of spatial acuity with increasing visual eccentricity. Human detail vision is limited to the fovea, the small central portion of the visual field subtending about 2° of visual angle. Beyond the fovea, spatial acuity drops rapidly as a function of eccentricity. For a part, this is due to the anisotropic distribution of photoreceptors in the retina. For instance, cone photoreceptors subserving photopic (day) vision are densely packed in the fovea where spatial acuity is high. With increasing eccentricity, rods subserving scotopic (night) vision become abundant, with less and less cones interspersed. Another factor adding to the limited peripheral acuity is that the responses of rods are usually pooled to increase sensitivity to light at the expense of spatial resolution; while the 1:1 correspondence in the fovea allows for maximal spatial resolution, the ratio of photoreceptors to ganglion cells can be as low as 130:1 in the periphery [27]. This implies that users might not be able to resolve and identify targets if they are located in the far periphery. Another peculiarity of peripheral vision is the so-called crowding effect [28-31]. Crowding refers to the phenomenon that the identification of objects in the visual periphery-is hampered if they are surrounded by similar objects. It has been suggested that crowding is caused by an inaccuracy in deploying spatial attention in the periphery, resulting in misbinding of features belonging to different objects [32].

Due to its visual design, the classical Matrix speller is inevitably affected by these peculiarities. The Matrix contains many symbols which are hard to allocate attention to in the periphery. One could up-size the symbols with increasing eccentricity to compensate for the decline of visual acuity, but this would then increase the crowding effect because the symbols get crammed together. The only way to scale-up element size and to counteract crowding at the same time is to decrease the number of symbols. Unfortunately, in the classical paradigm, this would leave the user with less degrees of freedom in communication. In contrast, both premises can be met with the Hex-o-Spell [33-35]. By means of a two-levels selection process, the Hex-o-Spell preserves a large vocabulary even though the number of symbols in the display is small. The original Hex-o-Spell consists of a central circle surrounded by six hexagons. Each hexagon represents a quintet of alphanumerical symbols. By means of motor imagery, the user rotates a central arrow and then chooses one of the hexagons. Upon choice, the symbols in the hexagon are expanded into the other hexagons and the user again uses mental imagery to choose the desired symbol. In this study, we adapt this two-level BCI design to an oddball paradigm and we compare its efficacy to the efficacy of the Matrix. Hex-o-Spell has some visually desirable properties. First, at each level, it displays only a few large symbols. This can prevent the detrimental effects of both declining spatial acuity and crowding. Second, the arrangement of hexagons is optimal with respect to crowding. Crowding is most serious if elements are placed on a line extending radially from the fixation point; it is minimal in configurations wherein elements are placed in a circular fashion, such as the hexagons of the Hex-o-Spell.

To investigate both modes of spatial attention (overt, covert) and both kinds of spellers (Hex-o-Spell, Matrix), we used a 2 × 2 within-subjects design. As benchmarks for the efficacy of each speller-attention pairing, we measured counting accuracy, ERPs, and classification performance in a copy-spelling task. In the ERP analysis, we investigated a number of different evoked and event-related components. In particular, in addition to the P3 component, we considered P1, N1, P2, and N2. P1, N1 and P2 are associated with automated stimulus processing that is affected by early attentional processes [36]. N2 is assumed to be related to the processing of deviant stimuli [37]. Despite the rather step-motherly treatment of these early components in earlier articles on ERP-based BCIs, there have been consistent reports that they are modulated in visual oddball tasks, first shown by [38] and corroborated by later studies [39,40]. Following the ERP analysis, we will present the results of offline classification using linear discriminant analysis (LDA) with shrinkage of the covariance matrix. Preliminary results of this study have been previously presented at a BCI workshop [41]. At a recent workshop, a similar study has been reported for the Matrix speller [42].

## Methods

### Participants

Thirteen participants (9 males and 4 females), aged 21-43 years (μ = 29.5) and naïve with respect to ERP-based BCIs, took part in the experiment. All had normal or corrected-to-normal vision and they received money for their participation. All participants gave written consent and the study was performed in accordance with the Declaration of Helsinki.

### Apparatus

**Figure 1:**
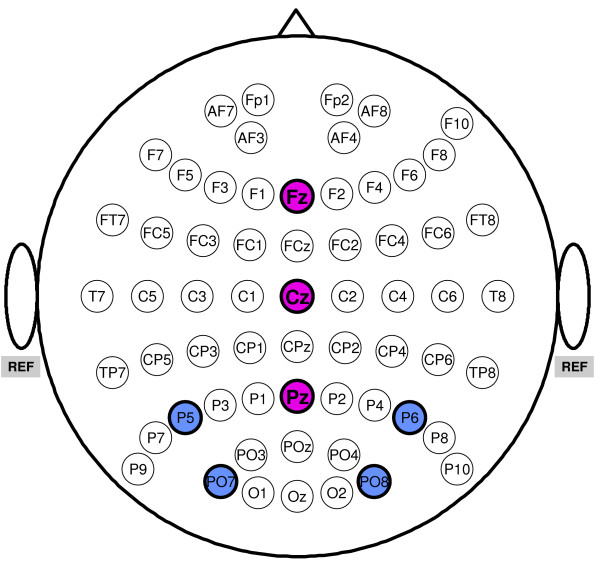
**Distribution of electrode sites on the scalp. Linked mastoids were used as reference**. The three midline sites (magenta) and the four parieto-occipital sites (blue) refer to electrode subsets employed in the statistical analyses.

EEG was recorded via a Brain Products (Munich, Germany) actiCAP active electrode system with 64 electrodes placed according to the international 10-10 system. Figure 1 depicts the distribution of electrodes on the scalp. Active electrodes were referenced to linked mastoids, using a forehead ground. All impedances were kept below 20 *k*Ω. EEG data were sampled at a rate of 1000 Hz and subjected to offline analysis. The bandpass of the hardware filter was 0.016-250 Hz. Concurrently with EEG recording, an Intelligaze IG-30 (Alea Technologies) eyetracker, sampling at 50 Hz, was used to register eye movements. Stimuli were presented on a 19" TFT screen with a refresh rate of 60 Hz and a resolution of 1280 × 1024 px^2^. The eyetracker was mounted underneath the screen.

### Stimuli

**Figure 2:**
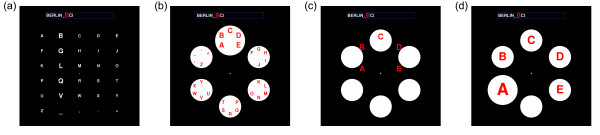
**Screenshots of the two visual spellers**. The current word was indicated in the box above the speller, and the current symbol was highlighted, (a) Symbol matrix. The column containing the target symbol **"B" **is intensified. (b) Hex-o-Spell, group level. The group containing the target symbol **"B" **(group "ABCDE") is intensified, (c) Transition phase. In a short animation, the symbols of the selected group are expanded onto the other discs. (d) Symbol level. The nontarget disc with the symbol "A" is intensified. The empty disc at the bottom is intended as a backdoor for returning to the group level in case the wrong group was selected.

Visual stimulation was achieved using two kinds of visual spellers, namely a Matrix speller (consisting of symbols arranged in rows and columns; Figure 2a) and an adapted version of the Hex-o-Spell (Figure 2bcd). The standard intensification type in ERP-based BCIs is contrast-enhancement ('lighting-up') of the symbols. However, this could potentially interfere with the deployment of attention in our covert attention condition, because peripheral low-contrast targets are susceptible to perceptual disappearance, a phenomenon known as Troxler-fading [43] which, moreover, is facilitated when the targets are flashed [44]. To reduce these effects, we defined intensification along the size dimension rather than the luminance dimension. This type of intensification enabled us to maintain maximum contrast for all symbols throughout a trial. Non-standard intensification types have been used earlier [45,46].

In the Matrix, symbols were arranged on a grid with a size of 500 × 500 px^2 ^(13.96° × 13.92°). In order to match the total number of symbols in the Hex-o-Spell (30), the speller comprised 6 rows and 5 columns (Figure 2a). Symbol height was 40 px (1.12°), or 65 px (1.82°) when intensified (an increase of 62.5%) with width depending on the particular symbol. Intensification was row-wise or column-wise. The Hex-o-Spell features selection as a two-stage process, wherein first a symbol group is selected (Figure 2b). Upon choice of a symbol group, the speller descends to the second level (Figure 2c), where the individual target symbol can be selected (Figure 2d). Note that, since there are 6 discs but only 5 symbols, one disc is empty; the purpose of the empty disc is to enable users to return to the top-level in case the wrong group has been selected. Discs had a size of 148 × 148 px^2 ^(4.15° × 4.14° of visual angle), or 200 × 200 px^2 ^(5.61° × 5.59°) when intensified (an increase of 35.1%). Unlike in the Matrix, discs in the Hex-o-Spell were intensified one by one. The discs were spatially arranged at the corners of an (invisible) hexagon with a diameter of 440 px (about 12.28°).

### Procedure

Participants were seated in a comfortable chair at a distance of about 60 cm from the screen, which is the optimal operational range for the eyetracker. Instruction was given both in written and verbal form. Participants were instructed to relax their muscles and to try to avoid eye movements during the course of a trial. After EEG preparation and calibration of the eyetracker, they completed a practice phase for the Matrix and for the Hex-o-Spell in the overt attention condition. After this, the experiment commenced and EEG was recorded for offline analysis. Participants engaged in a copy-spelling task, whereby they had to copy 5-6 letter words. There was a set of nine German words, chosen such that each letter in the English alphabet was covered 1-3 times. Each word was repeated 4 times (once for each subcondition). When a new word was introduced, it was shown on the screen prior to the start of the trial. Subsequently, a trial started with a 4-seconds auditory countdown, during which participants had time to identify the location of the target. The current word was always shown in a box above the speller with the current letter being highlighted (Figure 2). After the countdown, the intensification phase started, lasting for about 30s. The task of the participant was to silently count the number of intensifications of the target symbol. For the Matrix, 10 sequences were presented, whereby every row and every column was intensified exactly once in a single sequence (6 rows + 5 columns = 11 intensifications per sequence). The order of intensifications was pseudo-randomized as there had to be at least two intermittent intensifications before a particular intensification was repeated. Furthermore, to obtain meaningful behavioral data, some variation to the number of intensifications of a target was introduced. The sequences had a prequel and a sequel (both not used in the analysis) of 11 intensifications each, whereby intensifications were allowed to repeat. This added up to a total of 132 intensifications per trial. For the Hex-o-Spell, the sequences were evenly spread across the two hex levels. At the first level (group level), a symbol group had to be selected. Analogous to the Matrix, there were 10 sequences of 6 intensifications each, with prequels and sequels containing repetitions. At the second level (symbol level), the target symbol had to be selected, again with 10 sequences, and again preceded and followed by sequences with repetitions. Since, in the second level, there was also an empty disc, there was a total of 144 intensifications per trial. The number of target intensifications, however, was the same for both spellers.

For both spellers, the duration of a single intensification was 100 ms (or an equivalent of 6 frames). Stimulus onset asynchrony (SOA), that is, the time between the onsets of subsequent intensifications, amounted to 166 ms (10 frames). At the end of each trial, participants entered their count via the computer keyboard. The next trial commenced when participants pressed the enter key. In the overt attention condition, they had to fixate the target symbol or disc. In the covert attention condition, a central fixation dot was shown throughout the trial and participants had to strictly fixate the dot while counting the intensifications of the target. To assure proper fixation, eye movements were monitored online. If a fixation of a location other than the designated location (i.e., the target symbol or the fixation dot) was detected, a warning tone was presented and the trial was aborted; upon a button press, the trial was started again, using new intensification sequences.

The experiment was split into blocks of 3 words each, whereby breaks were given between the blocks. The order of the blocks was randomized, albeit with the constraint that each speller type was introduced first in the overt attention condition, not in the more difficult covert attention condition. The total number of blocks amounted to 2 (spellers) × 2 (attention) × 3 (unique blocks) = 12. The two spellers were implemented in the open-source BCI framework Pyff [47] and remote-controlled via Matlab. Both spellers are available on the Pyff website [48].

## Results

### Counting accuracy

**Figure 3:**
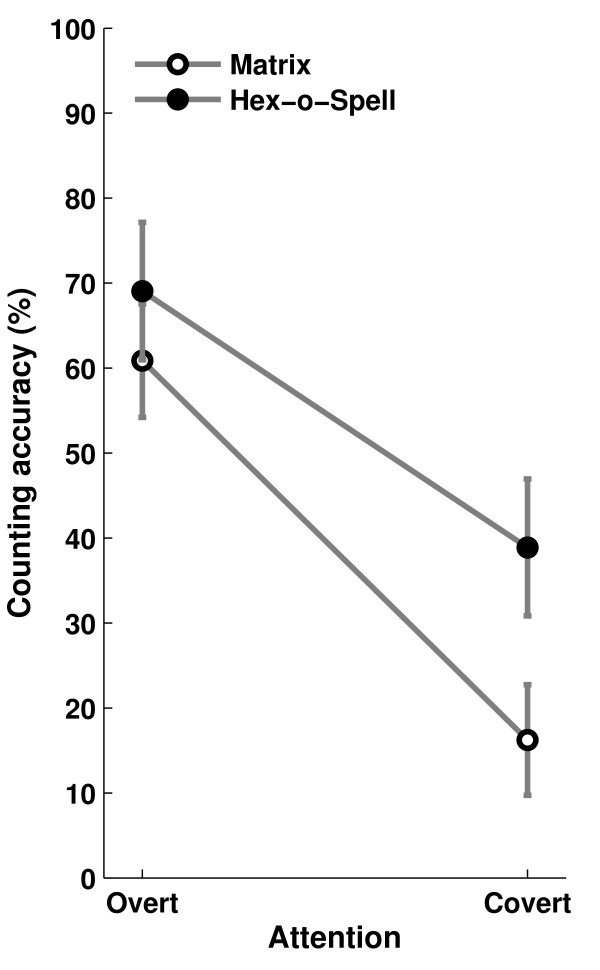
**Mean counting accuracy for the Matrix and the Hex-o-Spell in the two attention conditions**. Error bars show 1 SEM. Counting accuracy was higher for the Hex-o-Spell than for the Matrix, and higher for overt attention than for covert attention. The performance drop from overt to covert attention was more severe for the Matrix than for the Hex-o-Spell.

Due to technical difficulties, counting data was not available for one of the participants. For each of the remaining participants and for each subcondition, counting accuracy was determined. Accuracy was analyzed using a two-way repeated measures ANOVA, with factors Speller and Attention. Results are depicted in Figure 3. Overall, accuracy was higher for the Hex-o-Spell than for the Matrix (*Speller, F *= 41.42, *p *< .001), and accuracy was also higher for overt attention than for covert attention (*Attention, F *= 232.889, *p *< .001). In the overt attention condition, the difference between the spellers was significant, albeit small (paired-samples t-test, *t *= 3.441, *p *< .01). The drop in accuracy for covert attention condition compared to overt attention was more severe for the Matrix than for the Hex-o-Spell (*Speller *× *Attention, F *= 6.453, *p *< .05).

### Event-related potentials (ERPs)

**Figure 4:**
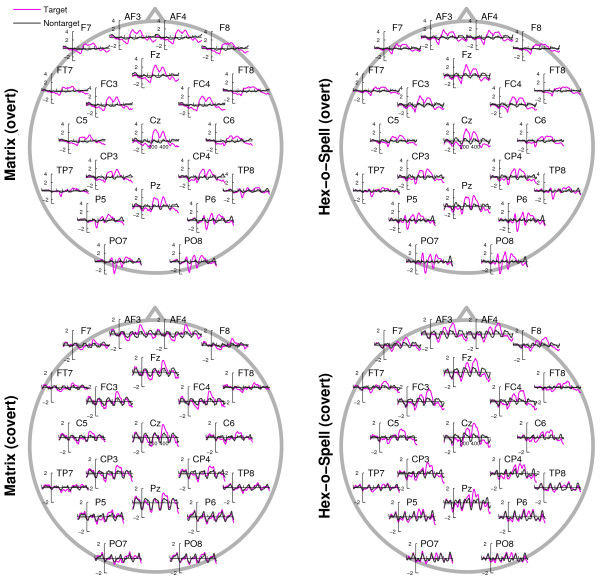
**Grand-average ERPs for each type of speller and each type of attention shown for 21 electrode sites**. Responses to targets are given in magenta, responses to nontargets in black. Epochs in the range [-170, 670] are shown. In the overt attention condition, for both the Matrix and the Hex-o-Spell, there are two clear positive peaks at ≈ 250 ms (P2) and ≈ 400 ms (P3), with a broad scalp distribution centered at Cz. A positivity at ≈ 120 ms (P1) and a sharp negativity at ≈ 150 ms (N1) preponderates at parieto-occipital sites. Also, a N2 component (≈ 300 ms) is evident at these sites. In the covert attention condition, the response to targets versus nontargets is less differential. In comparison to the overt attention condition, amplitudes are smaller for targets and larger for nontargets. Furthermore, in this condition, P2 is pronounced only in the Hex-o-Spell at fronto-central electrode sites. The pattern of P3 amplitudes is similar to the one encountered in the overt attention condition. Note that the y-axis scaling is different for the two attention conditions.

**Figure 5:**
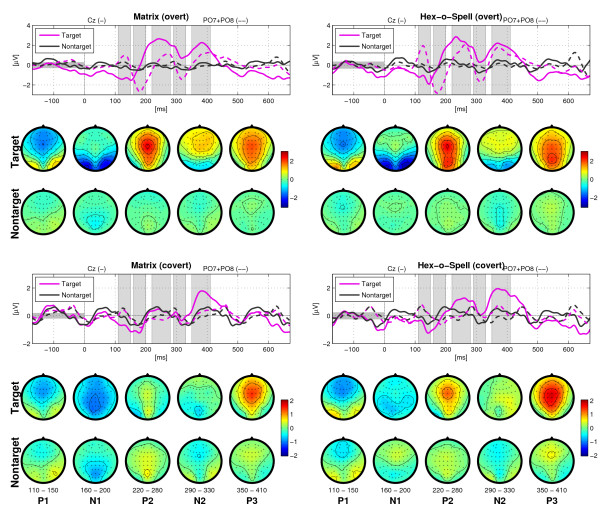
**Scalp distributions for the P1, N1, P2, N2, and P3 components**. For visualization purposes, the intervals (depicted above the component labels) chosen for the components are slightly narrower than the intervals used in the ERP analysis. The shaded areas in the ERP plots correspond to the intervals for which topographies are shown underneath. The solid line refers to electrode Cz and the dotted line refers to the average of electrodes PO7 and PO8. In the overt attention condition, it is particularly N1 and P2 that yield high amplitudes for target intensifications. In the covert attention condition, P3 yields the highest amplitudes.

For ERP analysis, EEG data was downsampled to 250 Hz and then divided into epochs ranging from -170 ms (roughly corresponding to one SOA) to 670 ms (roughly corresponding to four SOAs) relative to target intensification. Baseline correction was performed on basis of the 170 ms pre-stimulus interval. Owing to the short SOA, ERP components of subsequent intensifications were overlapping. This implies that preceding or immediately following intensifications would affect the ERP ascribed to a particular target, especially if these intensifications were also intensifications of the target. To prevent direct interference by previous or following intensifications of the target, only those epochs were considered wherein the interval to the previous or the next target intensification was at least 3 intensifications (or an equivalent of 500 ms). This was always the case for the Hex-o-Spell, but in the Matrix, targets could be repeatedly intensified when, for instance, the row and the column corresponding to the target were intensified in succession. Such epochs were not considered during ERP analysis. To also reduce contamination in the analysis of the nontarget epochs, only those nontarget epochs were considered wherein there were no targets for at least three preceding and at least the two following intensifications relative to nontarget onset. This kind of equalizing the ratio of conditions in preceding trials is of particular importance in case of overlapping and refractory effects of ERP components. Otherwise the biased influence of preceding trials makes the interpretation of ERP components disputable. Figure 4 shows the grand average scalp topography of the ERPs for both Matrix and Hex-o-Spell, and overt and covert attention. As shown in Figure 5, a number of prominent components can be distinguished. First of all, the P1, N1, and N2 components, preponderating at parieto-occipital sites. Second, P2 and P3, with central distributions elongated along the midline electrodes. Plots for each participant are given as additional file 1. Statistical analyses were performed using subsets of electrodes for which the components were most pronounced. For P1, N1, and N2, these were P5, P6, PO7, and PO8; for P2 and P3, midline electrodes Fz, Cz, and Pz were chosen (see Figure 1). Peak amplitude and peak latency were determined by picking the largest positive or negative peak within the intervals 80-150 ms (P1), 150-230 ms (N1), 210-290 ms (P2), 280-360 ms (N2), 350-440 ms (P3). When a particular component was not found within the according interval, the mean amplitude of the interval was taken as an approximate substitute. For each component, this yielded a four-factor design, *Speller *[Matrix, Hex-o-Spell] × *Attention *[overt, covert] × *Status *[target, nontarget] × *Electrode*, for analysis with a 4-way repeated measures ANOVA.

### ERP amplitudes

**Figure 6:**
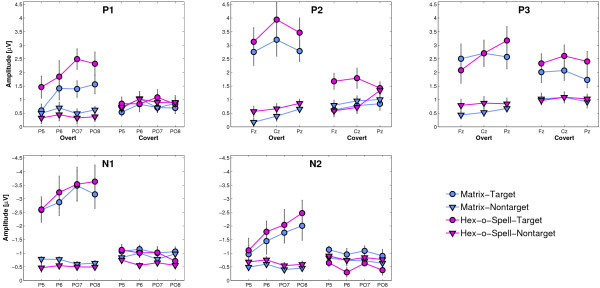
**Mean amplitudes for the positive components P1, P2, and P3, and the negative components N1 and N2**. Data is depicted separately for the Matrix and the Hex-o-Spell (colored in blue and magenta, respectively), and for overt and covert attention (left and right half of each plot, respectively). Error bars show 1 SEM. Note that the y-axis is reversed for negative components N1 and N2.

Mean peak amplitudes are depicted in Figure 6. Analysis of P1 amplitudes revealed larger amplitudes for targets than for nontargets (*Status, F *= 33.49, *p *< .001), larger amplitudes for the Hex-o-Spell than for the Matrix (*Speller, F *= 5, *p *< .05), and larger amplitudes for overt attention than for covert attention (*Attention, F *= 5.66, *p *< .05). P1 amplitude modulation was different for the two modes of attention (*Attention *× *Status, F *= 40.31, *p *< .001); there was significant modulation for overt attention (*Status, F *= 56.19, *p *< .001) but not for covert attention (*p *= .216). Furthermore, the modulation of amplitude in the overt attention condition was higher for Hex-o-Spell than for the Matrix (*Speller *× *Status, F *= 8.77, *p *< .01, and *Attention *× *Speller *× *Status *, *F *= 4.92, *p *< .05). The effect of *Electrode *(*p *= .055) and the other interactions were not significant.

Analysis of N1 amplitudes revealed larger amplitudes for targets than for nontargets (*Status, F *= 178.29, *p *< .001) and larger amplitudes for overt attention than for covert attention (*Attention, F *= 87.16, *p *< .001). Main effects of *Speller *(*p *= .403), *Electrode (p *= .698), and the other interactions were not significant. Like for P1, N1 amplitude modulation was different for the two modes of attention (*Attention *× *Status, F *= 119.07, *p *< .001); there was significant modulation for overt attention for both spellers (*Status, F *= 174.35, *p *< .001), but for covert attention modulation was significant for the Hex-o-Spell (*Status, F *= 9.14, *p *< .01) but not for the Matrix (*p *= .163).

P2 amplitudes were larger for targets than for nontargets (*Status, F *= 120.23, *p *< .001), larger for the Hex-o-Spell than for the Matrix (*Speller, F *= 10.34, *p *< .01), and larger for overt attention than for covert attention (*Attention, F *= 38.79, *p *< .001). P2 amplitude modulation was different for the two modes of attention (*Attention *× *Status, F *= 77.47, *p *< .001), and it was also different for the two different spellers (*Speller *× *Status, F *= 5.15, *p *< .05). In particular, modulation was stronger for overt attention than for covert attention, and stronger for Hex-o-Spell than for the Matrix. In the covert attention condition, there was no significant modulation of P2 amplitude for the Matrix (*p *= .354), but it was significant for the Hex-o-Spell (*Status, F *= 11.88, *p *< .01). The effect of *Electrode *(*p *= .18) and the other interactions were not significant.

N2 amplitudes were larger for targets than for nontargets (*Status, F *= 36.25, *p *< .001), and larger for overt attention than for covert attention (*Attention, F *= 15.75, *p *< .001). N2 amplitude modulation was different for the two modes of attention (*Attention *× *Status, F *= 38.71, *p *< .001), and these differences differed for the two spellers (*Attention *× *Speller *× *Status, F *= 4.25, *p *< .05). Amplitude modulation was higher for overt attention than for covert attention. In the covert attention condition, it was still significant for both the Matrix (*Status, F *= 4.53, *p *< .05) and the Hex-o-Spell (*Status, F *= 10.59, *p *< .01). However, for Hex-o-Spell, it was in the opposite direction (i.e., smaller amplitudes for targets than for nontargets). Although there was no significant effect of *Electrode *(*p *= .486), there were significant interactions, namely *Attention *× *Electrode *(*F *= 3.07, *p *< .05), and there was a significant three-way interaction *Attention *× *Target *× *Electrode, F *= 4.25, *p *< .05). The effect of *Speller *(*p *= .958) and the other interactions were not significant.

P3 amplitudes were larger for targets than for nontargets (*Status, F *= 129.52, *p *< .001). Amplitude modulation was higher for overt attention than for covert attention (*Attention *× *Status, F *= 7.75, *p *<.01). Main effects of *Attention *(*p *= .708), *Speller *(*p *= .109), *Electrode *(*p *= .491), and the other interactions were not significant.

In addition to these analyses, we also investigated the effects of attention and speller on difference amplitudes (i.e., ERP amplitude to target intensification minus ERP amplitude to nontarget intensifications). Because, as Figure 6 shows, not only target but also nontarget amplitudes were usually different across conditions, difference amplitudes give a better picture of the *magnitude *of amplitude modulation. For all ERP components under investigation, that is, P1, N1, P2, N2, and P3, we found that amplitudes are modulated more under overt attention than under covert attention (*Attention*, *F*-values 90.22, 160.81, 121.67, 43.91, and 13.65, respectively, with all *p*-values < .001). For positive components P1 and P2, we found overall stronger modulations for the Hex-o-Spell than for the Matrix (*Speller*, *F*-values are 19.64 and 8.09, respectively, *p *< .01). For the N2 and P3 components, amplitude modulations in the overt attention condition were not significantly different for the two spellers (*p*-values .6438 and .516, respectively), but they were stronger for the Hex-o-Spell than for the Matrix in the covert attention condition (*F *= 28.67, *p *< .001, and *F *= 5.23, *p *< .05, respectively). Regarding the N1 component, there was no effect of *Speller *on difference amplitude (*p *= .1).

### Classification

The ERP analysis in the previous section showed that there is a number of ERP components that is modulated by attention. A BCI operates by detecting these modulations and inferring whether a target or a nontarget was intensified. For offline classification, we used linear discriminant analysis (LDA) with shrinkage of the covariance matrix. A recent article on ERP analysis showed that shrinkage is a potent tool to counteract the bias encountered in settings with high-dimensional feature vectors and comparably small training sets, and it was shown to be at least as good as step-wise LDA (Blankertz B, Lemm S, Treder MS, Haufe S, Müller KR: Single-trial analysis and classification of ERP components - a tutorial, submitted). As said in the Procedure, there were three blocks of trials for each attention-speller pairing. For classification, the first block was taken as training set and the second and third blocks were taken as test set. EEG was downsampled to 100 Hz and baseline corrected using a 170 ms pre-stimulus interval. In contrast to the ERP analysis, all epochs were used for classification. The feature vector consisted of 55 spatial features × 7 temporal features = 385 spatio-temporal features. Temporal features were automatically extracted using a heuristic searching for peaks in the point-biserial correlation coefficient between targets and nontargets. A single binary (target versus nontarget) classifier was trained. To choose a symbol in the Matrix speller, the row (out of 6 rows) and the column (out of 5 columns) with maximum classifier outputs were selected, and the target symbol was given by their intersection [9]. For the Hex-o-Spell, the selection process was similar. At the group level, the group (out of 6 groups) with the highest classifier output was chosen, and at the symbol level, the symbol (out of 6 symbols) with the highest classifier output was chosen.

**Figure 7:**
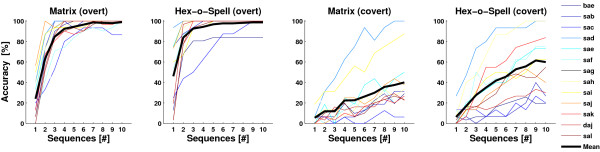
**Results of the classification as a function of number of sequences**. Thin colored lines indicate the performance of individual participants, the thick black line represents the mean in each subcondition. Accuracy approaches 100% using overt attention but is comparably low using covert attention. Hex-o-Spell outperforms the Matrix in both conditions.

Figure 7 depicts the results for each experimental subcondition. To interpret the results, notice that the chance level was 1/30 = 3.33% for the Matrix and 1/36 = 2.78% for the Hex-o-Spell. To test whether the differences between the subconditions are statistically significant, we applied a three-way repeated measures ANOVA with factors *Speller, Attention*, and *#Sequences*. Accuracy increases significantly with the number of intensification sequences (*#Sequences, F*(9,4) = 17.146). Higher accuracy is obtained with the Hex-o-Spell than with the Matrix (*Speller, F*(1,12) = 34.341), and higher accuracy with overt than with covert attention (*Attention, F*(1,12) = 146.321). The difference in performance between Matrix and Hex-o-Spell is larger for covert attention than for overt attention (*Speller *× *Attention, F*(1,12) = 8.443). Furthermore, because Matrix and Hex-o-Spell performance quickly reaches the ceiling for overt attention, the difference between the speller performances gets smaller, while, for covert attention, the difference between speller performances increases with the number of sequences (*Speller *× *Attention *× *#Sequences, F*(9,4) = 10.918).

**Figure 8:**
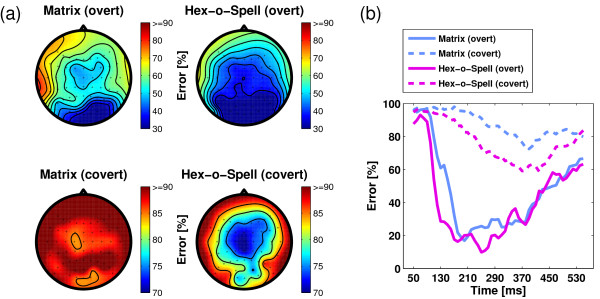
**Spatial and temporal distribution of discriminative information, (a) Classification errors obtained for each electrode separately are depicted as scalp topographies**. (b) Classification error for a single temporal feature, a 40 ms averaging window, for different positions of the center of the window.

To shed more light upon the spatial and temporal distribution of discriminative information, we re-run the classification, using the whole 10 intensification sequences with only spatial or only temporal features. Figure 8a shows the spatial distribution of discriminative information for the four subconditions. Classification was performed on one single electrode at a time, taking the time samples as temporal features. As the results show, the spatial pattern is determined by the mode of attention. In overt attention mode, most discriminative information is available at occipital and parieto-occipital electrode sites. In covert attention mode, centrally located electrodes yield the lowest classification error. Figure 8b shows classification performance using all electrodes but only one temporal feature (the mean of a 40 ms window). Classification error for overt attention is lowest in the range of about 180-250 ms, which is the range of the N1 and P2 components. In contrast, classification error for covert attention is high in this interval and descends only in the post-300 ms period, wherein the P3 occurs. Nevertheless, the curves for overt attention always remain below the curves for covert attention. In other words, the P3 component is also informative in overt attention mode, but it is less informative than the earlier ERP components.

## General discussion

Behavioral, neurophysiological, and classification indices unanimously attest an advantage (i.e., less errors, larger ERP amplitude, better classification) of overt attention over covert attention, and an advantage of Hex-o-Spell over Matrix. Using overt attention, spelling success is mainly based on visually evoked potentials (VEPs) measured at occipital and parieto-occipital sites. This confirms earlier conjectures [38,49] and it is also in accordance with [50] who showed that classifying on posterior electrodes in addition to the classical P3 sites improves classification performance. For a part, the comparably limited amount of information carried in the P3 component is due to the fast pace of the BCI used in the present study. In BCIs with longer SOAs, P3 components tend to be much more pronounced both in terms of amplitude and temporal extent [10,39]. In covert attention mode, classification is mainly based on the P3 component, but there is also a clear modulation of P2 amplitude for the Hex-o-Spell. In the face of these results, the term P300-BCI, which is often used in the literature, seems inadequate if not misleading. We advocate the use of the term *ERP-based BCI *to put emphasis on the fact that there is a multitude of ERP components that is affected by attention and that is exploited by classifiers. The rest of the General discussion addresses the role of (c)overt attention and discusses aspects that might be important in the design of visual spellers.

### Overt versus covert attention

If visual ERP-based BCIs are to have more than a shadowy existence in clinical practice, they have to form a viable alternative to eyetrackers. Currently, the detection of eye movements is quicker, easier, and more accurate than the detection of ERP modulations, and there are commercial plug-and-play eyetrackers tailored for users with motor deficiencies. Using eyetrackers, a spelling rate of 10 words per minute can be obtained with unimpaired eye movements. For ERP-based spellers, a recent study reported a spelling rate of 1.2 symbols per minute using a 6 × 6 Matrix speller with ALS patients [22], which is markedly lower. As a side note, notice that eyetrackers and BCIs do not need to be mutually exclusive systems in general. For instance, [51] demonstrated a hybrid system based on both eyetracking and BCI wherein targets were selected by eye gaze and an action was triggered via motor imagery.

In patients with impaired control of eye movements, however, reliable communication via eyetrackers can break down. But, since the neural systems underlying overt attention shifts and covert attention shifts are not identical [52], such patients might still be able to use covert attention. Our study shows that ERP-based visual spellers can be driven in both modes of attention, so they might replace or complement eyetrackers in these situations. Unfortunately, the accuracy obtained for covert attention in the present offline analysis is too low to be a viable means of communication. This implies that before ERP-based spellers are suitable for clinical practice, both classification [53] and visual design need to improve markedly. The latter point is addressed next.

### Visual speller design

Using covert attention, classification performance is low for both kind of spellers. For the Matrix, peak performance is about 40%. For the Hex-o-Spell, it is about 60%, which amounts to a relative increase of 50%. This illustrates that taking into account the peculiarities of peripheral vision can substantially boost performance. There is a number of aspects related to visual speller design that can be differentiated, namely the spatial arrangement of the elements on the screen, the visual properties of the elements, the intensification type, and the intensification sequence. We will address these points one by one.

As explicated in the Introduction, the deployment of spatial attention in the visual periphery is complicated by effects of crowding and decline of spatial acuity. Hex-o-Spell is less affected by these effects than the Matrix, because it features a small number of large elements instead of a large number of small elements, and because elements are arranged in a circular fashion, which has been shown to reduce crowding. In addition, the circular arrangement of elements around a central point in the Hex-o-Spell allows for a straightforward transition from a screen-centered representation to a body-centered representation in other modalities. For instance, [5,6] recently presented a spatial auditory paradigm wherein the user was located in the center of a ring of six loudspeakers. Users had to focus their auditory attention on one of the loudspeakers to choose a symbol. Due to the conceptual similarities between the visual Hex-o-Spell and the spatial auditory paradigm, users could probably switch more easily between the paradigms than when they had started with the Matrix.

With respect to the design of the individual elements, size matters. Not only can large elements be identified more easily in the periphery, there is also evidence that P3 amplitudes are positively correlated with stimulus intensity [54], which is in line with the fact that we found larger ERP amplitudes for the Hex-o-Spell than for the Matrix. Larger amplitudes might be of particular importance for clinical application, because an attenuation of ERP amplitudes is generally observed in ALS patients [14,55,56]. With respect to intensification type, there is a number of visual feature dimensions along which an intensification can be defined, for instance, luminance, size, form, orientation, color, and motion. In this study, we used size enhancement because it allows for maximum contrast both when the symbols are enhanced and when they are not. This is especially important when objects are presented in the visual periphery. Successful applications of other types of intensification have been reported in the literature, such as orientation, motion onset and illusory triangles [57-59]. Flipping the orientation of a rectangle located behind each symbol in a Matrix speller produced better classification results than the classical luminance intensification [58]. Furthermore, when intensifications were defined by motion onset, the positive and negative components in the 160-300 ms interval were discriminative for the task [57]. Interestingly, this interval was also the most informative interval in our overt attention condition. Actually, in contrast to luminance enhancement, both orientation flips and size enhancement imply that the contours of the symbols are displaced, which can give rise to a conscious percept of apparent motion. Stimulus intensity could be increased by using multiple features simultaneously for intensification; conversely, using different intensifications for different elements might increase their discriminability if a classifier is trained on each element separately.

With respect to intensification sequence, target-to-target distance (i.e., the number of nontargets between successive intensifications of the target) has been shown to affect the amplitude of the P3 component, with smaller amplitudes for more frequent targets [58,60]. This is in accordance with the fact that larger SOAs yield larger ERP amplitudes, because with larger SOAs the temporal separation between successive target intensifications increases. Hence, it is desirable to use target sequences wherein targets are not repeatedly intensified without nontargets in between. In the Matrix, the problem is inevitable because if a row intensification is followed by a column intensification (or vice versa), it is possible that the target lies at the intersection of these two intensifications. This is not the case for the Hex-o-Spell, where elements are individually intensified.

In the literature, there have also been other approaches to make visual spellers less dependent on eye movements [21] presented a speller whereby four different words were presented at the same spatial location in an alternating fashion. Both healthy users and ALS patients were able to communicate, which means that spatial attention is not necessary for visual spelling. The disadvantage of this paradigm, however, is that the sequential presentation of targets requires very long trials to maintain a large vocabulary. An alternative to sequential presentation of symbols might be simultaneous presentation at the same location. For instance, for the SSVEP paradigm, it was demonstrated that users can reliably choose between two superimposed dot patterns rotating in opposite directions [61] or between two superimposed gratings [62]. Again, however, the vocabulary is necessarily small with overlapping targets, which illustrates how difficult it is to reconcile independence from eye movements with high information throughput.

### Limitations

A few limitations warrant consideration. First, whether or not ALS patients with impaired eye movements can reliably employ covert spatial attention for BCI control has to be verified in clinical studies. Second, classification performance of the Matrix is compared to the performance of the Hex-o-Spell on basis of offline data. An online study would give a more accurate estimate of the performance of these two spellers.

## Conclusion

For patients with intact control of eye movements, eyetrackers are the device-of-choice, at least if information throughput is the evaluation criterion. The target group of ERP-based spellers therefore is patients with impaired eye movements. Our study shows that the performance of visual spellers deteriorates if one switches from overt to covert attention, but it also shows that performance can be increased using innovative spellers that take into account the peculiarities of peripheral vision.

## Competing interests

The authors declare that they have no competing interests.

## Authors' contributions

MT and BB designed the experiment and performed ERP analysis and classification. MT carried out the experiments, performed the statistical analysis, and produced the draft. All authors read and approved the manuscript.

## Supplementary Material

Additional file 1 **ERP topographies for single participants**. For each participant, the timing and the scalp topography of positive and negative ERP components is shown. Separate maps are shown for each of the experimental subconditions, that is, for each kind of speller (Matrix, Hex-o-Spell) and each kind of attention (overt, covert). Click here for file
