# Supplementary figures and images for: (C)overt attention and visual speller design in an ERP-based brain-computer interface
Source: Behav Brain Funct. 2010 May 28;6:28. doi: 10.1186/1744-9081-6-28 (PMC2904265; doi:10.1186/1744-9081-6-28)

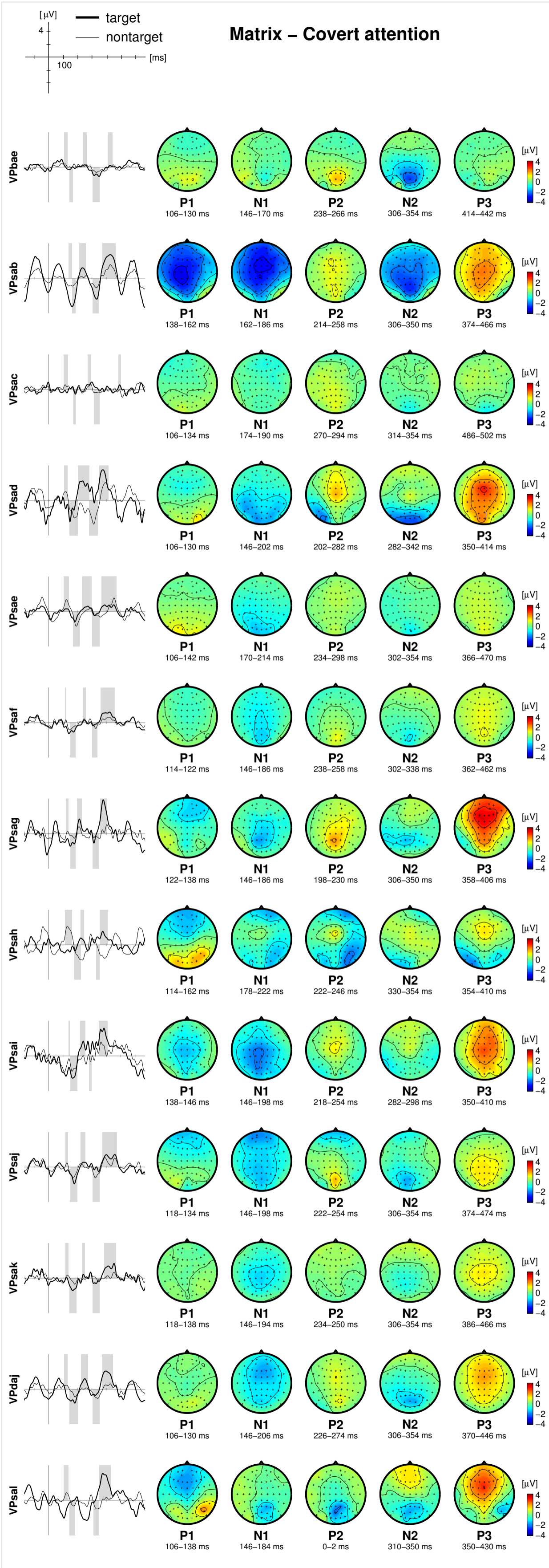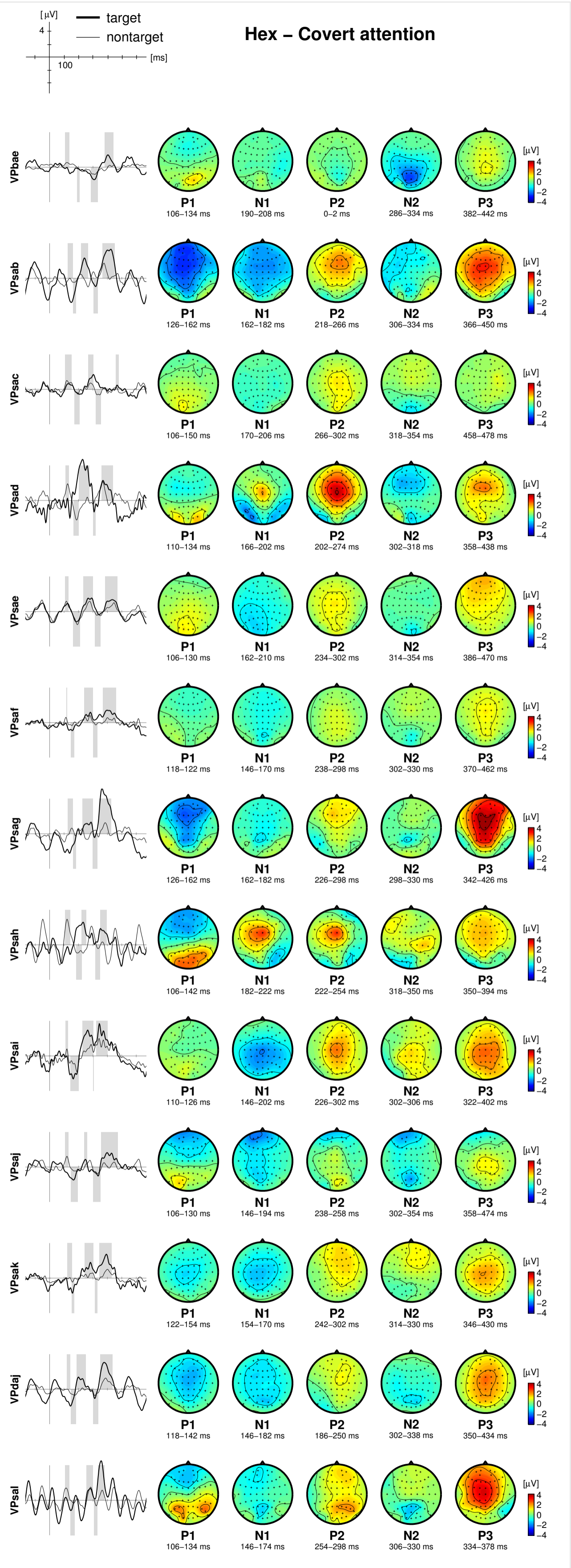

Supplement: Additional file 1 — ERP topographies for single participants. For each participant, the timing and the scalp topography of positive and negative ERP components is shown. Separate maps are shown for each of the experimental subconditions, that is, for each kind of speller (Matrix, Hex-o-Spell) and each kind of attention (overt, covert). [file 1744-9081-6-28-S1.PDF]
